# Personal Recovery in People With a Psychotic Disorder: A Systematic Review and Meta-Analysis of Associated Factors

**DOI:** 10.3389/fpsyt.2021.622628

**Published:** 2021-02-23

**Authors:** J. C. P. Leendertse, A. I. Wierdsma, D. van den Berg, A. M. Ruissen, M. Slade, S. Castelein, C. L. Mulder

**Affiliations:** ^1^Emergis Institute for Mental Healthcare, Kloetinge, Netherlands; ^2^Department of Psychiatry, Erasmus University Medical Centre, Rotterdam, Netherlands; ^3^Department of Clinical Psychology, VU University and Amsterdam Public Health Research Institute, Amsterdam, Netherlands; ^4^Research and Innovation, Parnassia Psychiatric Institute, The Hague, Netherlands; ^5^Department of Psychiatry, Haaglanden Medical Centre, The Hague, Netherlands; ^6^School of Health Sciences, Institute of Mental Health, University of Nottingham, Nottingham, United Kingdom; ^7^Lentis Research, Lentis Psychiatric Institute, Groningen, Netherlands; ^8^Faculty of Behavioural and Social Sciences, Clinical Psychology, University of Groningen, Groningen, Netherlands

**Keywords:** subjective recovery, person-oriented recovery, meta-analysis, psychosis, schizophrenia, personal recovery

## Abstract

**Background:** Personal recovery (PR) is a subjective, multidimensional concept, and quantitative research using PR as an outcome is rapidly increasing. This systematic review is intended to support the design of interventions that contribute to PR in psychotic disorders, by providing an overview of associated factors and their weighted importance to PR: clinical factors, social factors, and socio-demographic characteristics are included, and factors related to the concept of PR (organized into CHIME dimensions).

**Methods:** A systematic literature search was conducted from inception to March 2020. Quantitative studies that had used a validated questionnaire assessing the concept of PR were included. Mean effect sizes for the relationship between PR-scale total scores and related factors were calculated using meta-analyses. Sources of heterogeneity were examined using meta-regression tests.

**Results:** Forty-six studies, that used (a total of) eight PR measures, showed that in clinical factors, affective symptoms had a medium negative association with PR-scale total scores (*r* = −0.44, 95%CI −0.50 to −0.37), while positive, negative and general symptoms had small negative correlations. No association was found with neuro-cognition. Social factors (support, work and housing, and functioning) showed small positive correlations. Gender and age differences had barely been researched. Large associations were found for PR-scale total scores with the CHIME dimensions hope (*r* = 0.56, 95%CI 0.48–0.63), meaning in life (*r* = 0.48, 95%CI 0.38–0.58) and empowerment (*r* = 0.53, 95%CI 0.42–0.63); while medium associations were found with connectedness (*r* = 0.34, 95%CI 0.43–0.65) and identity (*r* = 0.43, 95%CI 0.35–0.50). Levels of heterogeneity were high, sources included: the variety of PR measures, variations in sample characteristics, publication bias, variations in outcome measures, and cultural differences.

**Discussion:** Most interventions in mental healthcare aim to reduce symptoms and improve functioning. With regard to stimulating PR, these interventions may benefit from also focusing on enhancing hope, empowerment, and meaning in life. The strength of these findings is limited by the challenges of comparing separate CHIME dimensions with questionnaires assessing the concept of PR, and by the high levels of heterogeneity observed. Future research should focus on the interaction between elements of PR and clinical and social factors over time.

## Introduction

Personal recovery (PR) is described as a highly individual process, whose definition is the subject of a debate that comprises a large and ever-growing body of literature. Several reviews have described PR in psychosis as either an idiosyncratic and non-linear process containing key elements (1–3), or as both process and outcome (4), or a multi-dimensional concept whose focus depends on individuals' experiences (5). Although consensus on the definition has not yet been reached (6), a widely endorsed theoretical basis for clinical and research purposes is offered by the conceptual framework of CHIME, the acronym for Connectedness, Hope, Identity, Meaning in life, and Empowerment (7).

When PR is considered an outcome, several validated questionnaires—such as such as the Recovery Assessment Scale (RAS) (8), the Questionnaire about the Processes of Recovery (QPR) (9), and the Mental Health Recovery Measure (MHRM)—can be used to measure PR (10). However, there is no gold standard (11), and a broad and multidimensional construct of PR can sometimes lead to ambiguous interpretations (12).

Quantitative research using PR as an outcome measure is nonetheless growing rapidly, and recently a call was made for more research into the ways in which interventions in specific groups may contribute to PR (6). A previous review indicated that PR improved over time when people are involved in recovery-oriented mental health treatment, especially when professionals collaborate with peer providers (13). Another recent review aimed to investigate the relationship between clinical and personal recovery, by performing a meta-analysis of the association between PR and (positive, negative, and affective) symptoms and functioning. Their findings suggested that clinical and personal recovery are only weakly associated, and that both need their own attention in treatment and outcome monitoring of people with psychotic disorders (14). The aim of the current study was to offer an overview of all factors associated with PR including social factors and demographics. Such an overview would add value to the development of interventions for improving PR in psychotic disorders, by giving direction to which elements to focus on.

The objective was therefore to systematically review and investigate the strength of the relationship between PR and associated factors in people with psychotic disorders. In our original study protocol we set out to look for associated factors in all quantitative studies assessing PR: interventions studies; cross-sectional studies; and longitudinal studies. However, when searching the literature, we came across two observations: firstly, only a very limited number of intervention studies were available that used PR as an outcome measure, and in these studies, no associations between PR-scale total scores and associated factors were described; and secondly, a large proportion of studies researched the association between PR-scale total scores and elements of PR itself (such as stigma and hope). In order to provide a complete reflection of the current state of literature, we decided to also include these factors related to the concept of PR. The CHIME dimensions were chosen as a way to organize these factors.

## Methods

### Literature Search

After pre-publishing the study protocol in the PROSPERO database (CRD42019121727), we conducted a literature search in Embase, PsychINFO, MEDLINE, Web of Science, Cochrane Central, and Google Scholar. To describe PR with a broad array of keywords, we used the following search terms: (subjective-, OR patient based-, OR consumer based-, OR person oriented-, OR personal recovery) in combination with the CHIME dimensions (connectedness, OR hope, OR identity, OR meaning, OR empowerment) in psychosis (psychosis, OR schizo-affective, OR schizophrenia) using a validated questionnaire of personal recovery (questionnaire, OR assessment, OR scale, OR instrument, OR inventory, OR psychometric). The review process was based on PRISMA guidelines.

Relevant articles were selected on the basis of the following inclusion criteria: peer-reviewed studies available in English, full-text, from inception to March 2020; DSM or ICD classifications of schizophrenia and other psychotic disorders (including affective psychotic disorders); both cross-sectional and longitudinal studies that used a validated questionnaire assessing the concept of PR and reported cross-sectional associations. Articles were excluded if they met the following exclusion criteria: severe mental illness (SMI) samples in which <65% of the study population had a psychotic disorder; use of item scores or subscale scores of personal recovery questionnaires, rather than total scores or validated short forms; pilot studies, feasibility studies, or implementation studies; and studies that performed secondary analyses on a sample that had already been included.

Retrieved publications were de-duplicated using EndNote X9 reference-management software. To identify studies that might meet our inclusion criteria, titles and/or abstracts were screened by the first rater (PL). Titles that were deemed relevant were screened independently by two members of the review team (PL and AR). Any disagreement on eligibility was resolved through discussion with a third author (DB). The full text of the remaining articles was screened (by PL) for factors associated with PR.

### Data Extraction

Data were extracted from the included studies. They included sample characteristics (sample size, percentage with a psychotic disorder); study characteristics (country, study design); the personal recovery measure used; all factors related to PR, including the measures used; and the corresponding effect sizes. Extracted factors were organized into four categories that were further subdivided into domains: (1) factors related to the concept of personal recovery (CHIME dimensions); (2) clinical factors (affective, positive, negative, and general symptoms; neuro-cognition); (3) social factors (support; work and housing; psychosocial functioning); (4) and factors not included in the meta-analysis due to the small number of studies: longitudinal findings, socio-demographic and other patient characteristics. For an overview of domains and corresponding factors, see the Supplementary Table 1.

To assess the strength of the cross-sectional relationship between factors and PR-scale total scores, we extracted correlation coefficients or corrected Beta-coefficients at baseline or T1 from the text or tables of included studies (15). A mean effect size was calculated for each domain. To ensure that each study contributed only one correlation per domain to the analysis, results per domain were averaged. For example, as stigma and self-esteem were both gathered under the CHIME dimension “identity,” they were averaged to obtain one overall correlation for PR-scale total score and identity. To ensure that all correlations within one factor-domain were interpreted in the same direction, coefficients were reversed where necessary. Following Cohen's convention, coefficients of 0.10, 0.30, and 0.50 were interpreted to demarcate small, medium, and large effects, respectively.

### Statistical Analyses

Metaforpackage in “R” was used to calculate mean effect-sizes per domain on the basis of random effects models using inverse-variance weighted Fisher's Z. Forest plots as visual summaries of the meta-analyses were inspected. Q-tests were conducted to test for evidence of heterogeneity, with I^2^ statistics as a method to quantify the level of heterogeneity. The source of heterogeneity was examined using meta-regression analyses, the predictors being PR measure and sample characteristics (100% psychotic disorder or less). To test for publication bias, Egger tests were used to detect funnel-plot asymmetry, but only if there were enough studies to perform this test. Sensitivity analyses were conducted to explore the effects of study quality.

### Study Quality

Study quality was independently assessed by two raters (PL and AW) using the NIH-Quality assessment tool for observational and cross-sectional studies (16). This tool covers fourteen study characteristics and was designed to focus on the key concepts for evaluating the internal validity of a study, including topics such as study objectives, sample selection, and adequate reporting. To facilitate sensitivity analysis—i.e., the exclusion of low-quality papers—the methodological quality of each study was rated as poor, fair or good. Statistical heterogeneity was reduced after excluding studies rated as poor; this mainly involved validation studies or studies in which personal recovery was not the primary outcome variable. However, point estimates and confidence intervals were not much affected by the exclusion (details of meta-analysis, sensitivity analysis, and tests of heterogeneity are available on request from the first author). To test for publication bias, Egger's method was used to detect funnel-plot asymmetry, but only if there were enough studies to perform this test. If statistically significant, the trim-and-fill method was used to explore the effect of publication bias.

## Results

### Study Characteristics

**Figure 1:**
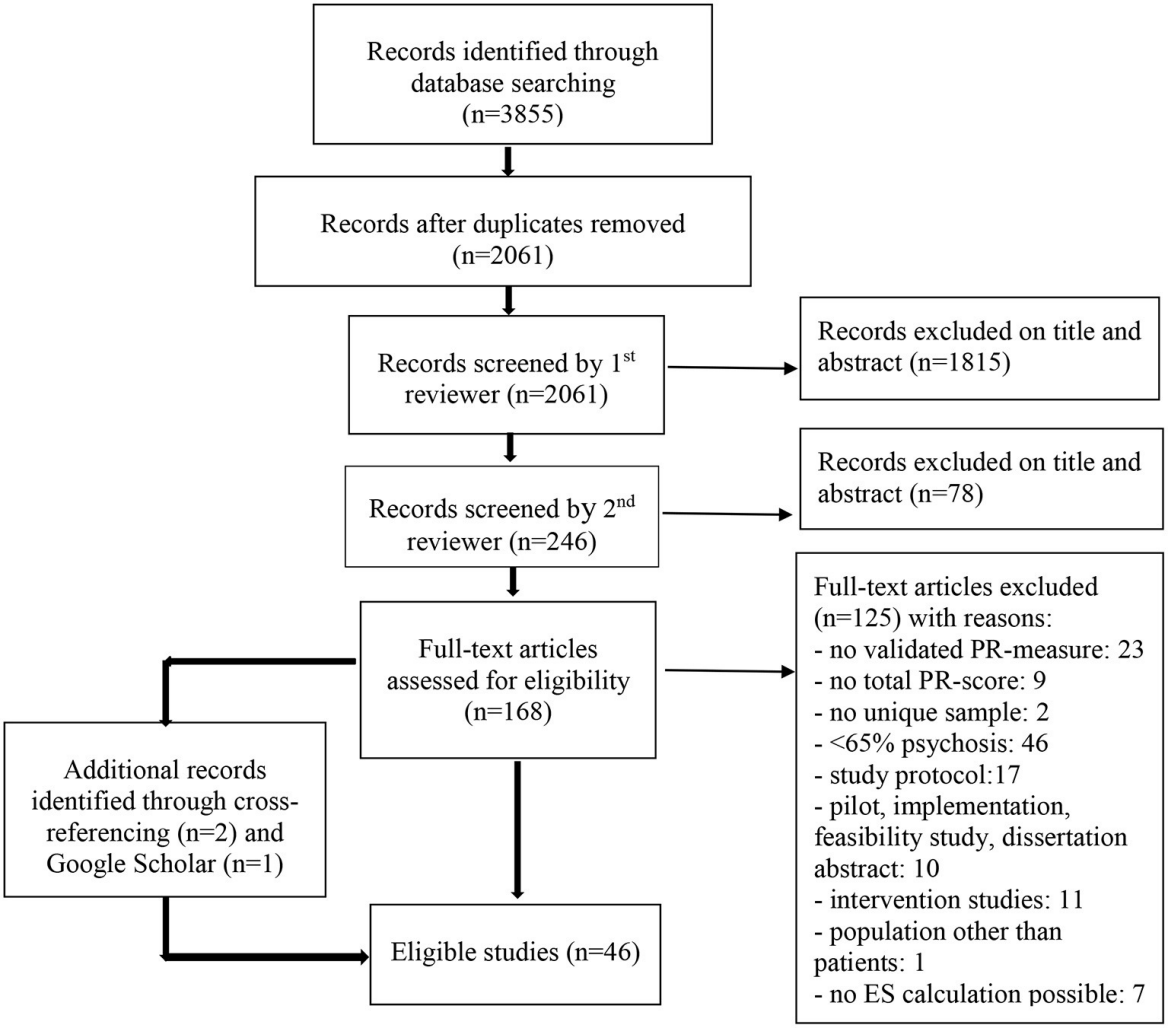
PRISMA flow diagram of studies including factors associated to personal recovery in psychosis (17).

Of the 2,061 papers found, 1,893 were excluded on the basis of abstract and title. The full text of the remaining 168 articles was assessed for eligibility, leading to the exclusion of 125 articles (reasons are shown in Figure 1). Google Scholar was consulted and reference lists of included studies were hand searched to check for missing studies, which resulted in three additional articles. This resulted in 46 studies that were eligible for inclusion in the review. Study characteristics are described in Table 1.

**Table 1:** Study characteristics of included studies (*n* = 46) in meta-analysis about associated factors with personal recovery in psychosis.

**References**	**N (% psychotic disorders)**	**Study design**	**Country**	**Personal recovery instrument**	**Associated factors—instruments**
Andresen et al. (10)	110 (100%)	Cross-sectional	Australia	RAS, MHRM	GAF, HoNOS, LSP, K10
Armstrong et al. (18)	795 (100%)	Cross-sectional	US	MHRM	LQoLI, CSQ-8, BPRS, MIRECC-GAF
Beck et al. (19)	122 (100%)	Cross-sectional	UK	QPR	HADS, BHS, SERS
Bhullar et al. (20)	65 (100%)	Longitudinal	UK, Canada	MARS	LCS
Boggian et al. (21)	216 (100%)	Cross-sectional	Italy	RAS	SESM, RSE, MANSA, HoNOS
Browne et al. (22)	404 (100%)	Longitudinal	USA	MHRM	QLS, SPWB
Brunet-Gouet et al. (23)	34 (100%)	Cross-sectional	France	STORI	V-MSEQ
Chan et al. (24)	181 (100%)	Longitudinal	China	RAS	SAPS, SANS, SOFAS, UPSA, MHC-SF
Chien and Chan (25)	300 (100%)	Cross-sectional	China	QPR	SQLS, SLOF, PSES
DeTore et al. (26)	404 (100%)	Cross-sectional	USA	MHRM	SCID-1, TLEQ
Erim et al. (27)	100 (100%)	Cross-sectional	Turkey	RAS	y/n question employment status
Espinosa et al. (28)	50 (100%)	Cross-sectional	Spain	RSQ	ISMI, BDI, BAI
Giusti et al. (29)	76 (100%)	Cross-sectional	Italy	RAS	BPRS, PANSS, PSP, RAVLT, Raven CPM, TMT, Weigl CFST, BCIS, IS
Gruber et al. (30)	138 (100%)	Cross-sectional	Germany	RSQ	WHOQOL-BREF, ISMI, SE, KK, ES
Guler and Gurkan (31)	180 (65%)	Cross-sectional	Turkey	RAS	PWS
Hasson-Ohayon et al. (32)	80 (100%)	Cross-sectional	Israel	RAS	SCC, ISMI, LRI
Hasson-Ohayon et al. (33)	107 (>80%)	Cross-sectional	Israel	RAS	ISMI, IS, SCC, RFQ, ADHS
Hicks et al. (34)	61 (100%)	Longitudinal	Australia	RAS	WAI-S, ADHS
Ho et al. (35)	204 (100%)	Cross-sectional	China	RAQ-7	HCCQ, MOSS-C-EIS, WHOQOL-BREF, WHOQOL-SRPBS, MSPSS-C, ASHS, ESCA, ISMI, RS, MS, SQLS, MDES
Jahn et al. (36)	169 (100%)	Cross-sectional	USA	MARS	BSI, PANSS (positive, negative)
Jorgensen et al. (37)	101 (100%)	Longitudinal	Denmark	RAS	PANSS
Kukla et al. (38)	113 (100%)	Cross-sectional	USA	RAS	PAM, PANSS, MS^1^, ASHS, IMR-S
Lavin and Ryan (39)	63 (67%)	Cross-sectional	Ireland	RAS	PWS, ASHS
Law et al. (40)	335 (100%)	Cross-sectional	UK	QPR	PANSS, Psyrats, BHS, SERS, CDSS, PSP
Lim et al. (41)	66 (100%)	Cross-sectional	Signapore	QPR	HHI, ISMI, ES, PANSS, CDSS, PSP, WHOQOL-BREF, RSWB
Mathew et al. (42)	80 (100%)	Cross-sectional	India	RAS	PANSS, SUBI, GAF
McLeod et al. (43)	89 (100%)	Cross-sectional	Australia	QPR	SEPRS, ISMI, involuntary treatment (y/n), contact recovered peers (high/low)
Morrison et al. (44)	122 (100%)	Cross-sectional	UK	QPR	HADS, SERS, IS, PANSS, MLCS, BACS
Mueser et al. (45)	399 (100%)	Cross-sectional	USA	MHRM	SS
O'Keeffe et al. (46)	171 (100%)	Longitudinal	Ireland	RAS	CD-RISC
Roe et al. (47)	159 (100%)	Cross-sectional	Israel	RAS	BPRS, GAF, MSPSS, S-SELAS, Mansa
Rossi et al. (48)	903 (100%)	Cross-sectional	Italy	RSQ	PANSS, PSP
Song (49)	592 (74.7%)	Cross-sectional	Taiwan	SRS	RPRS
Stainsby et al. (50)	50 (100%)	Longitudinal	UK	RSQ	IPQ-S, Mansa, LSP
Temesgen et al. (51)	263 (100%)	Cross-sectional	Ethiopia	QPR	PANSS, BHS, SSQ, ISMI, WHODAS, WHOQOL-BREF
Thomas et al. (52)	250 (100%)	Cross-sectional	USA	MARS	SSQ, ROSI, SEES < BSI, SFS
Van der Krieke et al. (53)	581 (100%)	Cross-sectional	Netherlands	RAS	WHOQOL-BREF, PANSS, SFS, CAN, RSQ
van Eck et al. (54)	105(76.6%)	Cross-sectional	Netherlands	MHRM	BPRS-E
Vass et al. (55)	80 (100%)	Longitudinal	UK	QPR	KSS, SERS, BHS, PANSS
Vass et al. (56)	59 (100%)	Cross-sectional	UK	QPR	KSS, ISMI, SERS, PANSS
Vogel et al. (12)	52 (100%)	Cross-sectional	Netherlands	MHRM	SSL-12-I
Williams et al. (57)	65 (100%)	Cross-sectional	Canada	RAS	BPRS, SAI, ISMI, BHS
Wood and Irons (58)	52 (100%)	Cross-sectional	UK	QPR	SS, OAS, SCS, PANSS (-positive), CDSS, BAI
Wood et al. (59)	79 (100%)	Cross-sectional	UK	QPR	SIMS-E, SIMS-P, ISS, SERS, BDI, BHS
Wright et al. (60)	62 (100%)	Cross-sectional	USA	QPR	MAI, TMT, BCIS, Metacognition (-appraisal task, detection task), WASI, UPSA, TUS, PANSS (neg, anxiety, depression), SSI-AE
Zizolfi et al. (61)	44 (100%)	Cross-sectional	Italy	RSQ	RS, MoCA, PANSS, LSP, SQLS

*ADHS, adult dispositional hope scale; ASHS, adult state hope scale; BACS, brief assessment of cognition in schizophrenia; BAI, beck anxiety inventory; BDI, beck depression inventory; BCIS, beck cognitive insight scale; BHS, beck hopelessness scale; BPRS, brief psychiatric rating scale; BPRS-E, BPRS expanded version; BSI, brief symptom inventory; CAN, camberwell assessment of needs; CD-RISC, Connor–Davidson resilience scale; CDSS, calgary depression scale for schizophrenia; CSQ-8, client satisfaction questionnaire; ES, Rogers empowerment scalel ESCA, exercise of self care agency; GAF, global assessment of functioning; HADS, hospital anxiety and depression scale; HCCQ, health care climate questionnaire; HHI, herth hope index; HoNOS, health of the nation outcome scales; IMR-S, illness management and recovery scale; IPQ-S, illness perceptions questionnaire for schizophrenia; IS, insight scale; ISS, internalized shame scale; ISMI, internalized stigma of mental illness; K10, Kessler psychological distress scale; KK, krankheitskonzept skala (illness concept scale); KSS, king stigma scale; LCS, life chart schedule; LSP, life skill profile; LQoLI, Lehman quality of life interview; LRI, life regard index; MANSA, Manchester assessment quality of life; MAI, metacognitive assessment interview; MDES, making decision empowerment scale; MHC-SF, mental health continuum-short form; MIRECC-GAF, mental illness research education and clinical centers version of the global assessment of functioning scale; MLCS, multidimensional locus of control scale; MOSS-C-EIS, emotional informational support scale of the medical outcome study social support survey chinese version; MS, mastery scale; MS^1^, morisky scale (of medication adherence); MoCA, montreal cognitive assessment; MSPSS, multidimensional scale of perceived social support; MSPSS-C, MSPSS–Chinese version; OAS, other as shamer scale; PAM, patient activation measure; PANSS, positive and negative syndrome scale; PANSS-positive, PANSS positive symptoms subscale; PANSS-negative, PANSS negative symptoms subscale; PANSS-GP, PANSS general psychopathology subscale; PSES, perceived elf-efficacy scale; PSP, personal and social performance scale; Psyrats, psychotic symptom rating scales; PWS, psychological well-being scale; QLS, quality of life scale; Raven CPM, Raven colored progressive matrices; RAVLT, Rey auditory verbal learning test; RFQ, functioning questionnaire; ROSI, recovery oriented system indicator; RS, resilience scale; RSE, Rosenberg self-esteem; RSQ, recovery style questionnaire; RSWB, Ryff scales of well-being; SAI, schedule for assessing insight; SANS, scale for the assessment of negative symptoms; SAPS, scale for the assessment of positive symptoms; SCC, self-concept clarity scale; SCID-1, structured clinical interview for axis I DSM–IV disorders; SEES, self-efficacy scale; SCS, social comparison scale; SE, self-esteem scale by rosenberg; SEPRS, self-efficacy for personal recovery scale; SERS, brief self-esteem rating scale; SESM, empowerment scale; SFS, social functioning scale; SIMS-E, semi-structured interview measure of stigma- experienced stigma subscale; SIMS-P, SIMS- perceived stigma subscale; SLOF, specific level of functioning scale; SSI-AE, Schizotypal symptom inventory - anomalous experiences; SSL-12-I, social support list 12 interactions; SOFAS, social and occupational functioning assessment scale; SPWB, scales of psychological well-being; SQLS, schizophrenia quality of life scale; SS, stigma scale; S-SELAS, social and emotional loneliness scale—short version; SSQ, social support questionnaire; SUBI, subjective well-being inventory; TLEQ, traumatic life events questionnaire; TMT, trail making test; TUS, time use survey; UPSA, University of California, San Diego, Performance-Based Skills Assessment; V-MSEQ, versailles metacognitive strategies evaluation questionnaire; WAI-S, working alliance inventory—short form; WASI, Wechsler abbreviated scale of intelligence; Weigl CFST, Weigl's color form sorting test; WHODAS, world health organization disability assessment schedule; WHOQOL-BREF, world health organization quality of life—BREF version; WHOQOL-SRPBS, WHOQOL spirituality religion and personal belief scale*.

### Personal Recovery

In total, eight PR measures were used: Recovery Assessment Scale (RAS) (*n* = 18 studies) (8); Questionnaire about the Process of Recovery (QPR) (*n* = 15) (9); Mental Health Recovery Measure (MHRM) (*n* = 6) (10); Recovery Style Questionnaire (RSQ) (*n* = 5) (62); Maryland Assessment of Recovery (MARS) (*n* = 3) (63); Recovery Attitudes Questionnaire (RAQ-7) (*n* = 1) (64); Stages of Recovery Scale (SRS) (*n* = 1) (65); and Stages of Recovery Instrument (STORI) (*n* = 1) (66). One study used both the MHRM and RAS.

### CHIME Dimensions

Large positive associations with PR-scale total scores were found for hope (*r* = 0.56, 95% CI = 0.48–0.63, *p* < 0.001); meaning in life (*r* = 0.48, 95% CI = 0.38–0.58, *p* < 0.001); and empowerment (*r* = 0.53, 95% CI = 0.42–0.63, *p* < 0.001). Medium positive associations with PR were found for connectedness (*r* = 0.34, 95% CI = 0.26–0.42, *p* < 0.001); and identity (*r* = 0.43, 95% CI = 0.35–0.50, *p* < 0.001). Inspection of the forest plots and Q-tests for all domains suggested heterogeneity between studies. I^2^ tests indicated high levels of heterogeneity for all CHIME dimensions, which ranged from 73.9 to 93.5% (see Table 2A). Meta-regression tests indicated differences caused by the use of the RAS as measure of PR in all the analyses of CHIME dimensions, although most differences were small and did not reach significance. One exception was the association between PR and empowerment, where RAS significantly increased the positive association (0.34, 95%CI = 0.19–0.48, *p* < 0.001). Due to the small number of studies, however, these results should be interpreted with caution. Heterogeneity can also be attributed to sample characteristics. Meta-regression indicated that studies using samples with 100% psychotic disorders (rather than SMI with >65% psychotic disorders) reduced the association between PR and meaning in life (−0.38, 95%CI = −0.76 to −0.00, *p* = 0.05); PR and empowerment (−0.24, 95% CI = −0.65–0.17, *p* = 0.25); and PR and connectedness (−0.14, 95%CI = −0.34–0.05, *p* = 0.16). One exception was the increased association between PR and identity (0.21, 95%CI = −0.14–0.55, *p* = 0.24). None of these results reached significance. In addition, results for connectedness and empowerment were based on a small number of studies. In the studies investigating the association between PR-scale total scores and hope, the regression test for funnel-plot asymmetry indicated publication bias (*z* = 3.970, *p* < 0.001). For the results of the meta-analyses and tests of heterogeneity, see Table 2A. For forest plots, see the Supplementary Table 2.

**Table 2A:** Meta-analysis results and tests of heterogeneity for CHIME dimensions.

**CHIME dimensions**	**K**	**Mean ES**	**95%CI**	**Homogeneity (Q, df)**	**I^**2**^**
Connectedness	7	0.34	0.26–0.42	Q(*df* = 6) = 26.5846, *p* = 0.0002	73,9%
Hope	12	0.56	0.48–0.63	Q(*df* = 11) = 47.6065, *p* < 0.0001	77.1%
Identity	25	0.43	0.35–0.50	Q(*df* = 24) = 189.3599, *p* < 0.0001	85.7%
Meaning	17	0.48	0.38–0.58	Q(*df* = 16) = 192.1816 *p* < 0.0001	93.5%
Empowerment	60	0.53	0.42–0.63	Q(*df* = 5) = 21.5806, *p* = 0.0006	74.2%

*K, number of studies in de analysis; Mean ES, pooled effect size of the individual studies; Q-test, test for homogeneity, significant Q-tests indicate heterogeneity; I^2^, quantification of heterogeneity, 25% indicating low heterogeneity, 50% moderate, and 75% high heterogeneity*.

### Clinical Factors

A medium negative association with PR-scale total scores was found for affective symptoms (*r* = −0.44, 95% CI = −0.50 to −0.38, *p* < 0.001). Small negative associations with PR-scale total scores were found for positive symptoms (*r* = −0.22, 95% CI = −0.28 to −0.15, *p* < 0.001); negative symptoms (*r* = −0.22, 95% CI = −0.28 to −0.16, *p* < 0.001); and general symptoms (*r* = −0.26, 95% CI = −0.37 to −0.15, *p* < 0.001). I^2^ scores ranged from 65.5 to 90.0%, indicating that the proportion of the total variance explained by heterogeneity was moderate to high. There was an indication of publication bias in the association between PR-scale total scores and positive symptoms (*z* = −2.27, *p* = 0.023). The trim-and-fill method showed a relatively small reduction (0.3) of the correlation estimate. Meta-regression analysis indicated that the use of the QPR increased the negative association between PR-scale total scores and all symptom domains (affective, positive, negative, and general symptoms). No association with PR-scale total scores was found for neuro-cognition (*r* = 0.05, 95%CI = −0.12 to 0.22, *p* = 0.536). Although only a moderate degree of heterogeneity between studies on PR and neuro-cognition was found (*I*^2^ = 72%), examination of the forest plot showed that there were outliers in both directions. For the results of the meta-analyses and tests of heterogeneity, see Table 2B. For forest plots, see Supplementary Table 2.

**Table 2B:** Meta-analysis results and tests of heterogeneity for clinical factors.

**Clinical factors**	**K**	**Mean ES**	**95%CI**	**Homogeneity (Q, df)**	**I^**2**^**
Affective symptoms	13	−0.44	−0.50 to −0.37	Q(*df* = 12) = 32.964, *p* = 0.0010	65.5%
Positive symptoms	19	−0.22	−0.28 to −0.15	Q(*df* = 18) = 61.598, *p* < 0.0001	75.3%
Negative symptoms	18	−0.22	−0.28 to −0.16	Q(*df* = 17) = 63.386, *p* < 0.0001	70.2%
General symptoms	15	−0.26	−0.37 to −0.15	Q(*df* = 14) = 124.783, *p* < 0.0001	90.0%
Neurocognition	7	−0.05	−0.12 to 0.22	Q(*df* = 6) = 18747, *p* < 0.0046	72.1%

### Social Factors

Small positive associations were found between PR-scale total scores and support (*r* = 0.28, 95% CI = 0.20–0.36, *p* < 0.001); work and housing (*r* = 0.23, 95% CI = 0.00–0.44, *p* = 0.046); and psychosocial functioning (*r* = 0.31, 95% CI = 0.21–0.41, *p* < 0.001). There was a high degree of heterogeneity between studies in the association between PR-scale total scores and psychosocial functioning (*I*^2^ = 92.5%) that was not attributable to variation in sample characteristics or type of PR measure. In the association between PR-scale total scores and support, the degree of heterogeneity was low (*I*^2^ = 35.2%). The meta-regression test indicated that the positive association was reduced by the use of the QPR. The number of studies investigating the association of PR and work and housing was too small for meaningful interpretation of analyses of heterogeneity. For results of the meta-analyses and tests of heterogeneity, see Table 2C. For forestplots, see Supplementary Table 2.

**Table 2C:** Meta-analysis results and tests of heterogeneity for social factors.

**Social factors**	**K**	**Mean ES**	**95%CI**	**Homogeneity (Q, df)**	**I^**2**^**
Support	5	0.28	0.22–0.36	Q(*df* = 4) = 6.059, *p* < 0.1948	35.2%
Work and housing	3	0.23	0.00–0.44	Q(*df* = 2) = 11.788, *p* < 0.0028	82.4%
Psychosocial functioning	20	0.31	0.21–0.41	Q(*df* = 19) = 224.287, *p* < 0.0001	92.7%

### Other Factors

Few studies reported on effects of socio-demographic and patient characteristics. Five studies investigated the relationship between age and PR-scale total scores (19, 29, 36, 39, 56), only one of which found a small (negative) association (r = −0.23, *p* < 0.05), indicating that older age was related to lower PR (19). One study investigated the relationship between gender and PR-scale total scores (56), and another investigated the relationship between education and PR-scale total scores (36); both found negligible differences. Other studies reported no significant differences in PR-scale total scores for years of illness (29), medication adherence (38), contact with recovered peers (43), or involuntary treatment (43). However, PR-scale total scores were found to be positively associated with physical health (*r* = 0.30, *p* < 0.001) (35). A negative association was found for PR-scale total scores with a diagnosis of comorbid PTSD (r = −0.13, *p* = 0.01) (26). PR-scale total scores were also found to be negatively associated with type of diagnosis (schizophrenia or bipolar disorder) (*r* = −0.41, *p* < 0.01), indicating that having a non-affective psychotic disorder is related to lower PR-scale total scores as compared to an affective psychotic disorder (56).

Few studies reported on longitudinal findings. Three studies investigated the relationship between duration of untreated psychosis (DUP) or untreated illness (DUI) and PR-scale total scores over follow-up periods ranging from over 2 years, to 10 or 20 years (20, 22, 46). Overall, results were inconclusive although some negative associations were reported. In one study a non-affective psychotic disorder was found to be related to lower PR-total scores over 20 years as compared to an affective psychotic disorder, while lifetime substance abuse was not related (56). Another study reported on the associations of PANSS-subscales and PR-scale total scores at baseline and after 3, 6, and 12 months (37): no statistically significant correlations were found for the Cognitive scale, whereas only the Emotional Discomfort Component showed medium to strong negative correlation coefficients at all four time points. No associations were found between PR-scale total scores and illness perception or quality of life over 2 years (50). Some other studies reported on positive correlation coefficients over a 6 month period for well-being (24), working alliance (34), and perceived stigma (55).

## Discussion

The aim of this systematic review and meta-analysis is to provide an overview of factors associated with PR-scale total scores in people with a psychotic disorder. In our original study protocol we planned to include intervention studies, however the literature search revealed that the few available intervention studies did not report cross-sectional associations with PR. Furthermore, we found that a large proportion of studies researched the association between PR-scale total scores and elements of PR itself. We decided to include these as well, and to organize them into CHIME dimensions. Unsurprisingly, considering the overlap, the associations between PR-scale total scores and the CHIME dimensions were medium to large. This overlap is confirmed by the fact that some studies investigated PR-scale total scores in relation to CHIME dimensions in order to assess the convergent validity of a PR measure (21, 25, 30, 31, 40, 41, 49).

Large positive associations with PR-scale total scores were found for meaning in life, empowerment and hope, whereas medium associations were found for identity and connectedness. This is in line with qualitative studies, which indicated that PR from the point of view of people with psychotic disorders can be defined in terms of faith, hope, agency and spirituality (3).

As determinants of PR-scale total scores, only affective symptoms appeared to have a medium negative association. All other factors showed either small negative associations (positive, negative, and general symptoms), or small positive associations (support, work and housing, and psychosocial functioning). No association was found with neuro-cognition, and the relatively small number of studies that investigated sociodemographic characteristics found no uniform effect for age.

However, interpretation of these associations was impeded by heterogeneity between studies in almost all domains. This heterogeneity had several sources, one being the variety of PR measures. As PR is a highly subjective concept, variation in PR measures is inevitable. We found that the QPR was linked more strongly to the symptom domains, while the RAS was linked more strongly to the CHIME dimensions. The RAS is known to have a particular emphasis on hope and self-determination (8); this may offer one explanation for the fact that its use reinforces the positive association between CHIME dimensions and PR-scale total scores. Another explanation for the high levels of heterogeneity may lie in the influence of heterogeneity in the study sample (SMI with >65% psychosis, rather than samples with 100% psychotic disorder). There was also evidence for publication bias in the associations between PR-scale total scores and the domains positive symptoms and hope, although the trim-and-fill method indicated only a small effect of publication bias on the associations.

Variation in the independent variables may also have contributed to the level of heterogeneity. This was illustrated, for example, in the domain of neuro-cognition, which consisted of neuropsychological tests, observer-rated neurocognitive functioning, and self-rated metacognitive functioning. Similarly, in the domain of psychosocial functioning, GAF appeared to be more weakly associated with PR-scale total scores compared to other scales of psychosocial functioning. Previous research suggested that this was due to the fact that GAF comprises both functioning and symptoms (14). Cultural values are also likely to play a role in a subjective construct such as PR (12, 67). Between different countries, for example, large differences have been found in the associations between PR-scale total scores and meaning in life (21, 31, 39, 47).

### Limitations

We used the CHIME model (7) in our literature search as a framework for PR. However, there are also other frameworks for PR, such as the SAMHSA statement, which offers 10 recovery components as essential mediators of recovery (68). Although, like CHIME, this aims to enhance recovery in mental health, we chose CHIME because it is widely endorsed (6), and because one of the purposes of the framework is to provide keywords for use in systematic reviewing (7).

The literature search limited to peer-reviewed studies available in English, which may not represent all of the evidence and may have introduced a language bias.

When certain factors were combined, detailed information on individual factors may have been lost. For example, this may have happened when averaging the correlation of stigma, and self-esteem with PR-scale total scores, in order to obtain one effect size per study for the CHIME dimension “identity”.

Studies in which < 65% of the study population had a psychotic disorder were excluded from the analysis. This percentage was based on expert opinion only, since a clear cut-off point could not be found in guidelines or previous research. To further objectify this decision, sample characteristics (100% psychotic disorder or less) was included as a predictor in the meta-regression analysis, but results indicated no significant differences because of variation in sample characteristics.

### Implications for Future Research

This review is consistent with previous research showing that, in psychotic disorders, symptoms and PR are weakly related, with affective symptoms showing medium associations and all other symptom domains showing small associations (14). Social factors and the (partly overlapping) CHIME dimension “connectedness” showed weaker associations with PR-scale total scores than expected. Previous qualitative research indicated that support, social inclusion and recovery-oriented practices (which are known to focus on these themes), are the main facilitators of PR (2, 6). In line with this, recent research on recovery-oriented interventions suggested that PR is mutually beneficial to functional domains (e.g., employment, education, housing) and social domains (e.g., social functioning and support, and community integration), meaning that gains in one domain can contribute to gains in another (69). However, quantitative studies in people with psychotic disorders have paid relatively little attention to the association of PR with these domains. In fact, as the three factors included in the domain of work and housing all concerned employment, we could not examine the independent effect of housing on PR. We therefore suggest that future research should focus on the relationship between PR and a greater number of social factors (e.g., support in employment and housing, and community integration) and between PR and connectedness (e.g., working relationship, social network, and level of perceived support).

Our meta-analysis focused on cross-sectional correlations since few longitudinal studies were included. However, longitudinal findings of PR in SMI were in line with our own findings, suggesting that without an explicit focus on recovery-oriented principles (e.g., personal goals, needs and strengths and a collaborative working relationship), mental health services are unlikely to affect PR (13). Nevertheless, more research is needed into the interaction between elements of PR and clinical and social factors over time. In line with previous research (12–14), we also suggest that future research would benefit from consensus on a PR measure.

### Implications for Clinical Practice

A multifactorial approach to improving PR in psychosis appears to be indicated. Many treatments for psychotic disorder patients focus on reducing psychotic symptoms and improving functioning. Previous research underlined the weak associations between elements of clinical recovery (except for affective symptoms) and PR (14). Likewise, our study suggests only weak associations with social factors like support, work, and feeling connected, which were expected to be important domains of PR in psychotic disorder. Only three CHIME dimensions, i.e., meaning in life, empowerment, and hope, showed strong associations with PR-scale total scores, in contrast to the other two dimensions: connectedness and identity. Therefore, we suggest on the basis of the cross-sectional results of our study that if we wish to enhance PR, treatments should focus on affecting the elements of PR itself. PR is a multidimensional construct, and most PR interventions focus on more dimensions. However, meaning in life, empowerment, and hope seem to be the dimensions to focus on. In addition, symptoms and their associated distress should be approached with evidence-based psychological treatment (70, 71), with a particular emphasis on negative affect. Little is known about how these factors might influence each other: for example, having hope may reinforce the effectiveness of treatment in reducing distress associated with symptoms of psychosis, and the reduction of distress associated with symptoms may reinforce having hope for the future.

There are empirically validated interventions for each of these PR elements. Meaning in life for example is supported by narrative-enhanced cognitive therapy (72), and can involve post-traumatic growth (73). With regard to subjective quality of life (SQOL)—a concept pertaining to the CHIME dimension “meaning in life”—previous research also stressed the association with negative affect in people with psychotic disorders, proposing that treatment plans for improving SQOL should focus on feelings of guilt, insecurity or anxiety (74). Meaning in life is also about spirituality. A recent review emphasized the significant role of spirituality in the lives of mental-health service users, and the importance for professionals not only of being aware of spirituality, but also of supporting it (75). Empowerment is an increasing focus for clinician-delivered interventions (76) and peer-delivered support (77); it is also a focus for the movement toward rights-oriented mental-health systems (78). Finally, peer support work is an established and highly researched approach to supporting hope (79, 80). On the basis of their review of longitudinal findings of PR in SMI, Thomas et al. (13) suggested that PR should be promoted by including themes such as self-management skills and self-determination as standard components of mental health services. However, implementing recovery-oriented practices into routine mental health is challenging (6). Implementation is influenced by organizational values and priorities, and culture. One illustration of this is the fact that well-designed interventions such as REFOCUS increase PR only when they are properly implemented (81, 82).

## Conclusions

Overall, in view of the tautological question of comparing PR with PR elements, and also of the high levels of heterogeneity between studies, we speculate with some caution that when one seeks to improve PR in psychosis, an emphasis on enhancing meaning in life, empowerment and hope, in addition to symptom reduction and improvement of functioning, might lead to better outcome. Future research should focus on the interaction between elements of PR and clinical and social factors, e.g., how hope and changes in symptoms due to effective treatment influence each other over time, and more research is needed into the relationship between PR and social factors.

## Data Availability Statement

The original contributions presented in the study are included in the article/supplementary material, further inquiries can be directed to the corresponding author.

## Author Contributions

JL, AR, and DB conducted the literature search. JL and AW extracted data and performed data analyses. All authors contributed to drafting the manuscript, contributed to the conception and design of the study, and approved the final version.

## Conflict of Interest

The authors declare that the research was conducted in the absence of any commercial or financial relationships that could be construed as a potential conflict of interest.
