# Supplementary material for: Personal Recovery in People With a Psychotic Disorder: A Systematic Review and Meta-Analysis of Associated Factors
Source: Front Psychiatry. 2021 Feb 23;12:622628. doi: 10.3389/fpsyt.2021.622628 (PMC7940758; doi:10.3389/fpsyt.2021.622628)
Supplement: Supplementary file 1 [file Table_1.DOCX]

Table 1. Categories, domains, and corresponding factors to be analysed for their associations with Personal Recovery scales

| Category | Domain | Factors |
| --- | --- | --- |
| CHIME dimensions | Connectedness | Loneliness, support (- of peers, - of professionals, - of services), working alliance |
|  | *Hope & optimism of the future* | Hope, hopelessness |
|  | *Identity* | Stigma (internalized -, perceived -, experienced-), shame (internalized -, external -), resilience, clinical insight, illness (-management, -perception, -attitude), self (-management, -esteem, -directedness, -clarity, -efficacy, -responsibility), treatment motivation, social rank |
|  | *Meaning in life* | Quality of life, well-being, meaning in life, spirituality |
|  | *Empowerment* | Empowerment, locus of control |
| Clinical factors | *Affective symptoms*  *Positive symptoms*  Negative symptoms | Depression, anxiety, emotional discomfort, excitement, negative emotions, suicide ideation  Positive symptoms, including positive subscales (PANSS, BPRS), disorganization, thought disturbance, anomalous experiences  Negative symptoms, including negative subscales (PANSS, BPRS), anergia |
|  | *General symptoms* | PANSS-GP subscale (preference), PANSS total scores (if no PANSS subcales are provided) or BPRS total scores |
|  | *Neurocognition* | Neurocognitive tasks, cognitive symptoms, cognitive insight, meta-cognition |
| Social factors | *Support* | Environmental satisfaction, social relations, social support |
|  | *Work & housing* | Occupational functioning, residential status |
|  | *Psychosocial functioning* | Functioning (psychosocial-, daily-, social-), level of (-activation, -disability), psychosocial (-needs, -symptoms) |
| Sociodemographic factors |  | Age, gender, level of education |
| Patient-characteristics  Longitudinal findings |  | Medication adherence, contact with recovered vs. not-recovered peers, involuntary treatment (y/n), diagnosis (BPD/SZ), PTSD, physical health, years of illness  DUP, DUI, well-being, working alliance, PANSS, substance abuse, illness perception, quality of life, perceived stigma |
| *Note:* PANSS, Positive and Negative Symptom Scale; BPRS, Brief Psychiatric Rating Scale; PANSS-GP, PANSS-General Psychopathology subscale; BPD, Borderline Personality Disorder; SZ, Schizophrenia; PTSD, Post Traumatic Stress Disorder; DUP, Duration of Untreated Psychosis; DUI, Duration of untreated Illness; RSQ | | |
