# Supplementary material for: Personal Recovery in People With a Psychotic Disorder: A Systematic Review and Meta-Analysis of Associated Factors
Source: Front Psychiatry. 2021 Feb 23;12:622628. doi: 10.3389/fpsyt.2021.622628 (PMC7940758; doi:10.3389/fpsyt.2021.622628)

Table 2a. Forest plots of CHIME domains and Personal Recovery


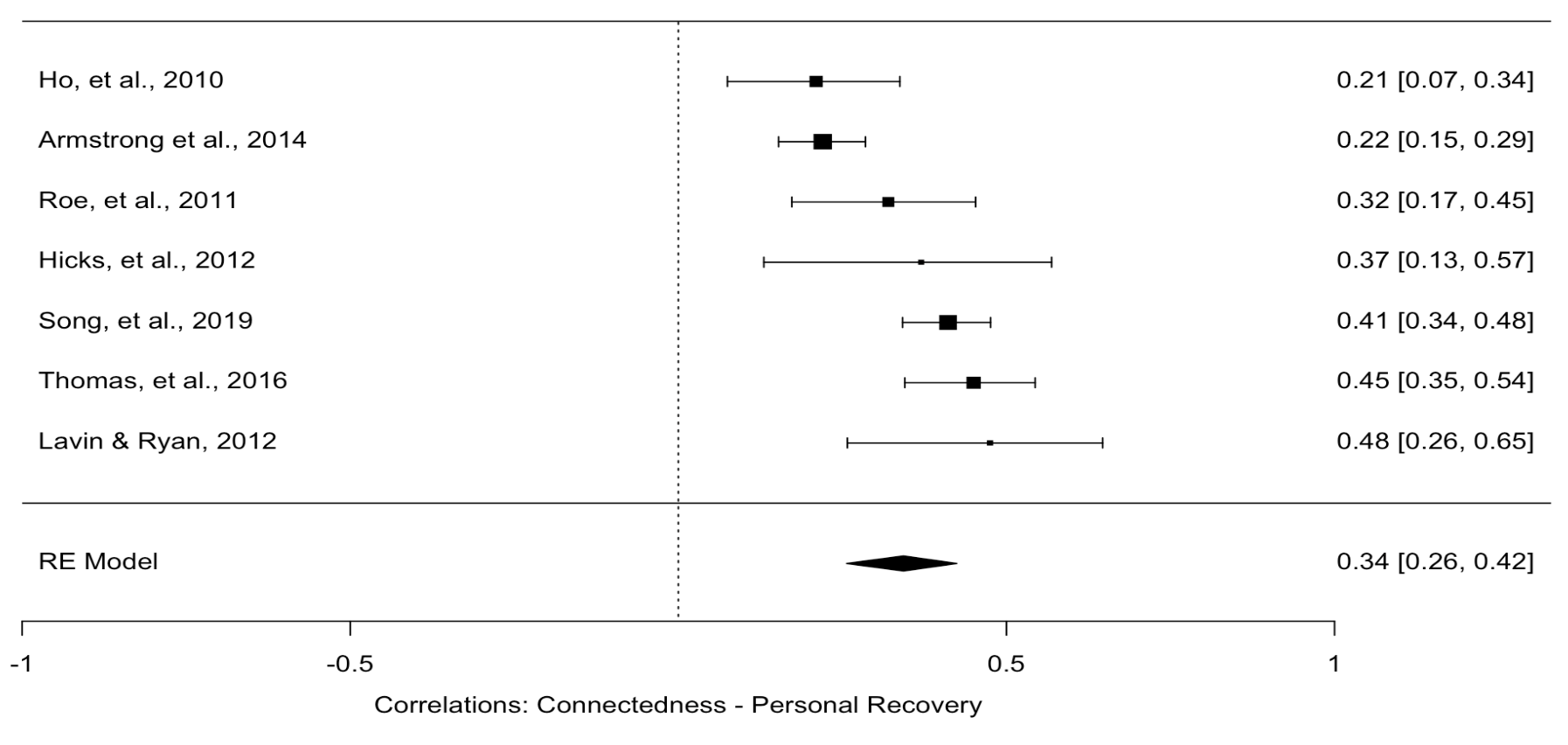


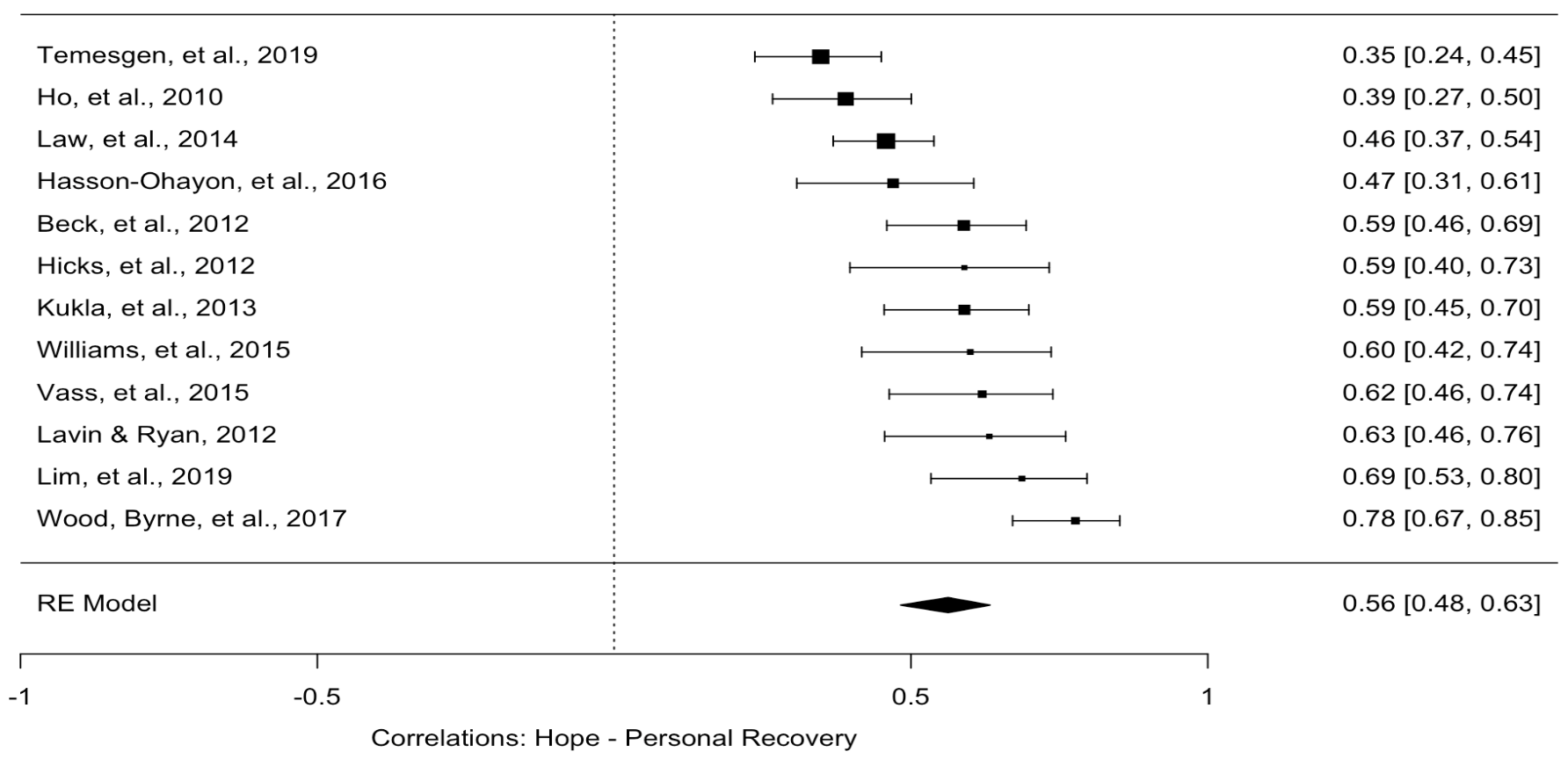


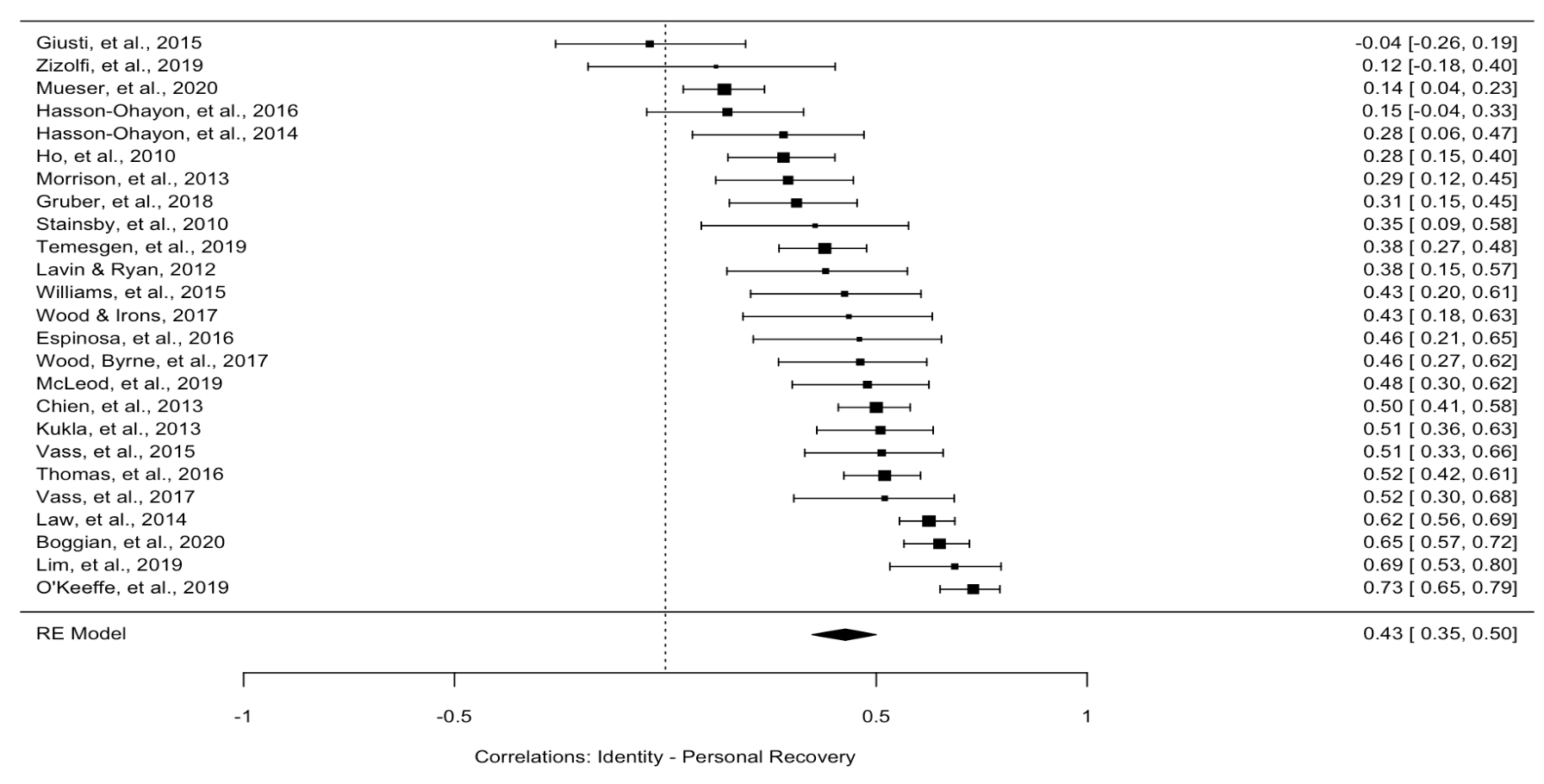


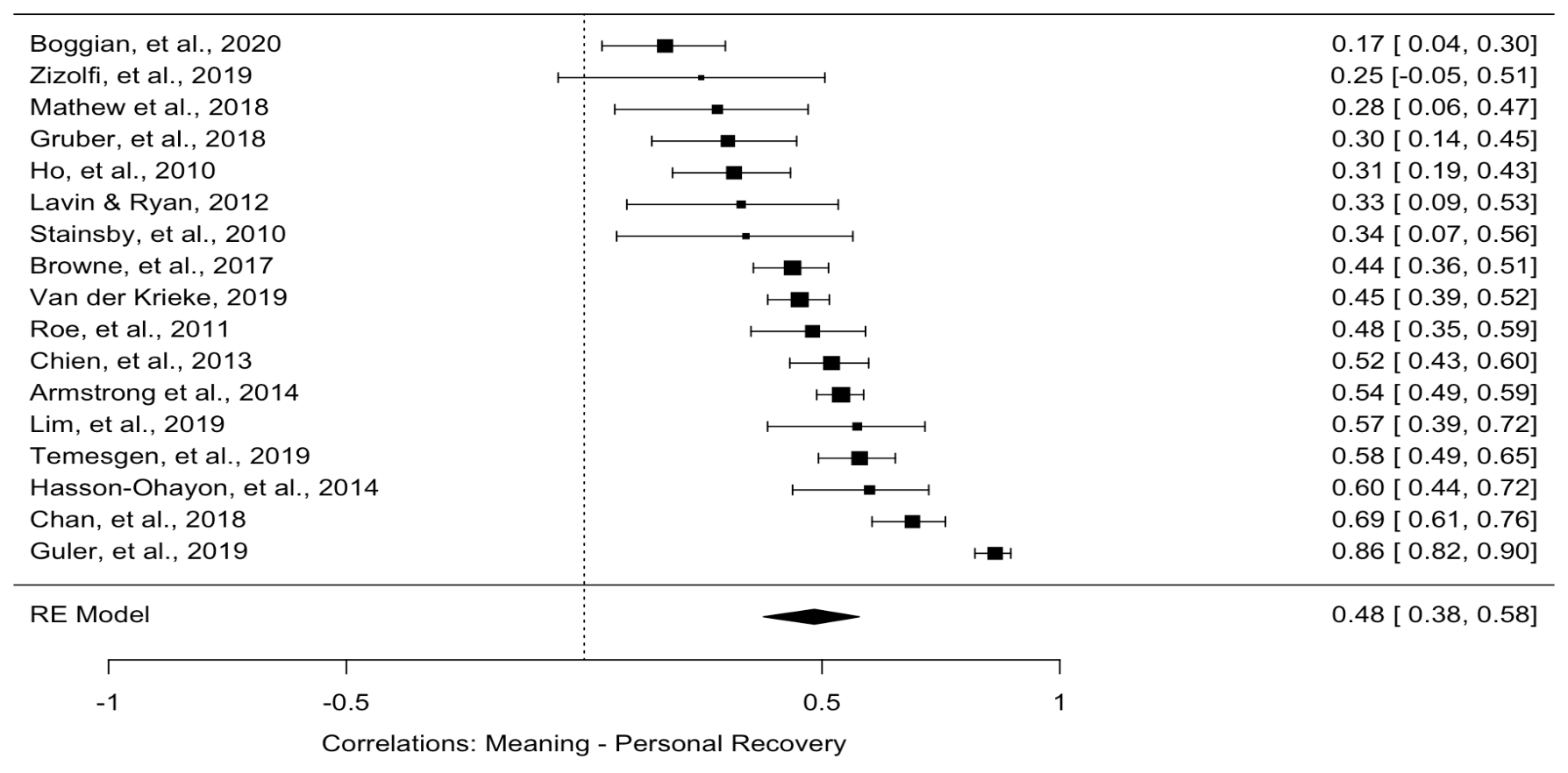


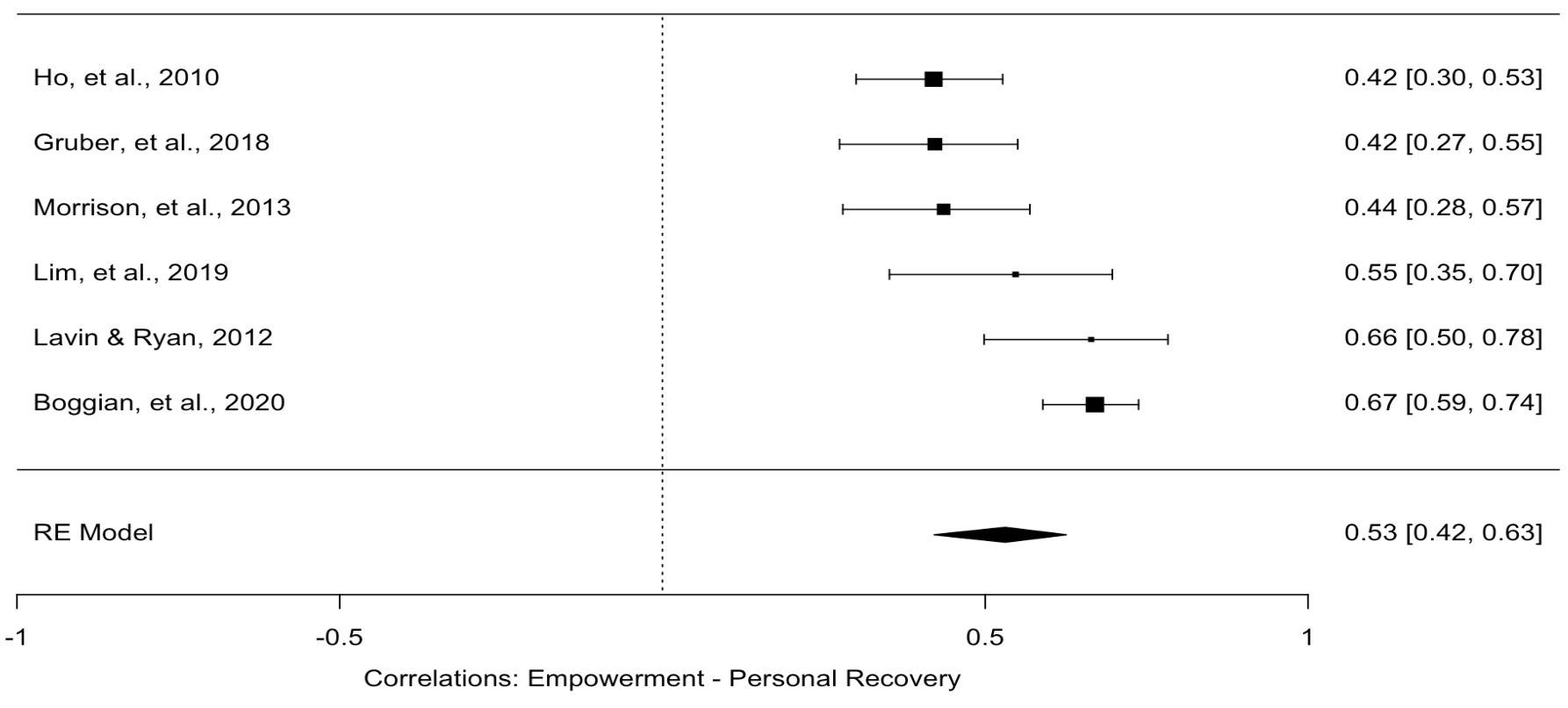


Table 2b. Forest plots of Clinical factors and Personal Recovery


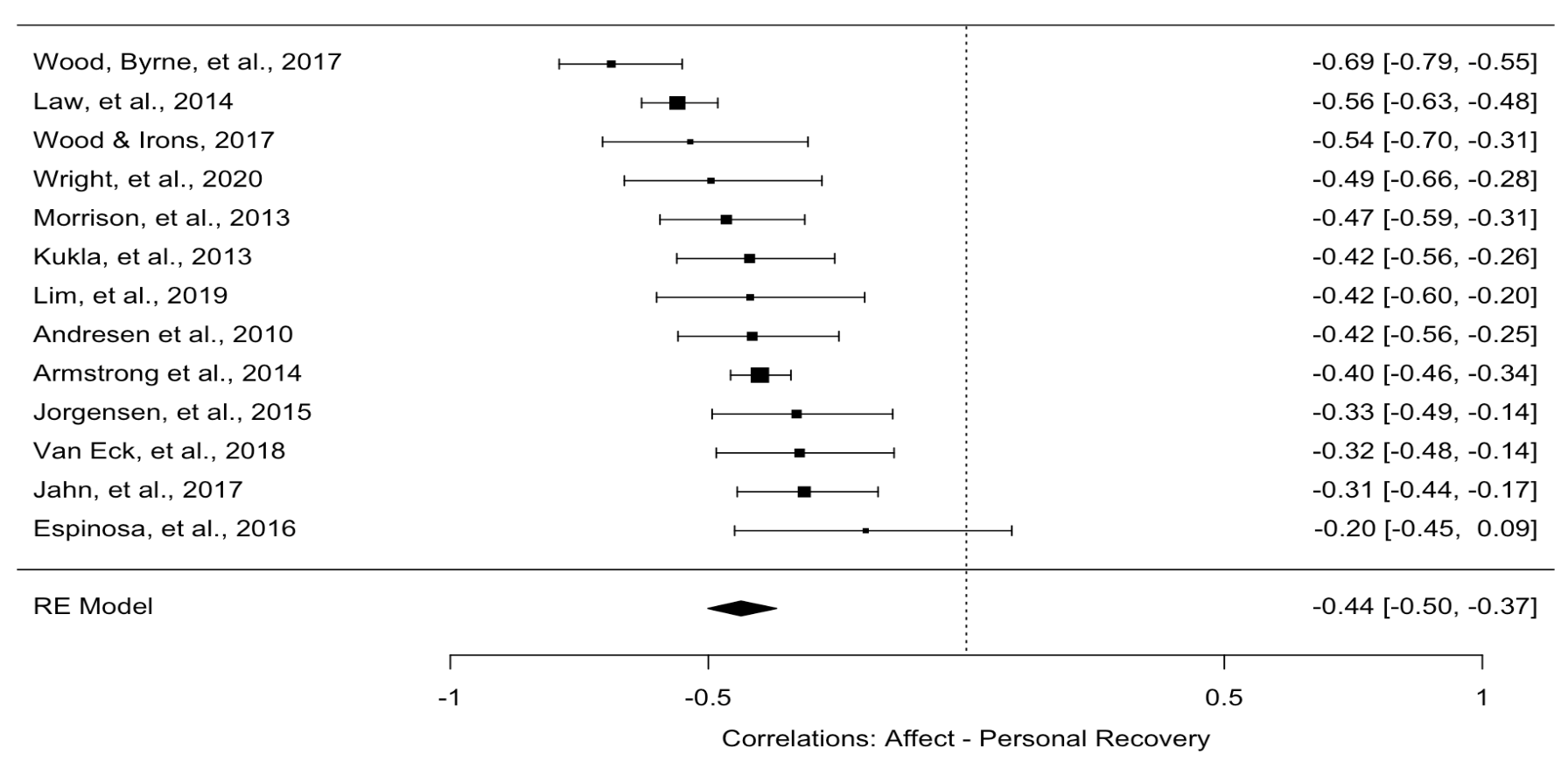


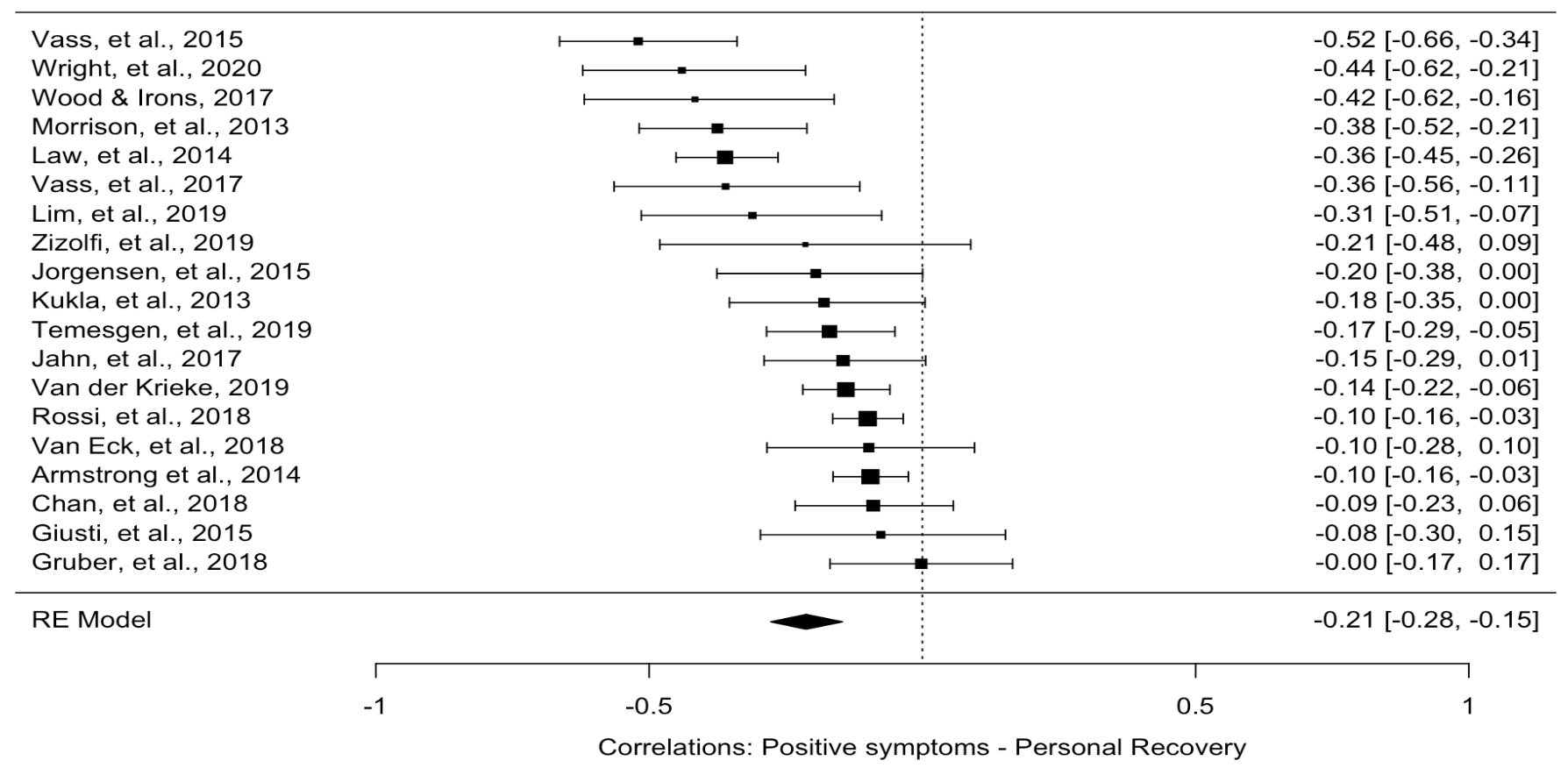


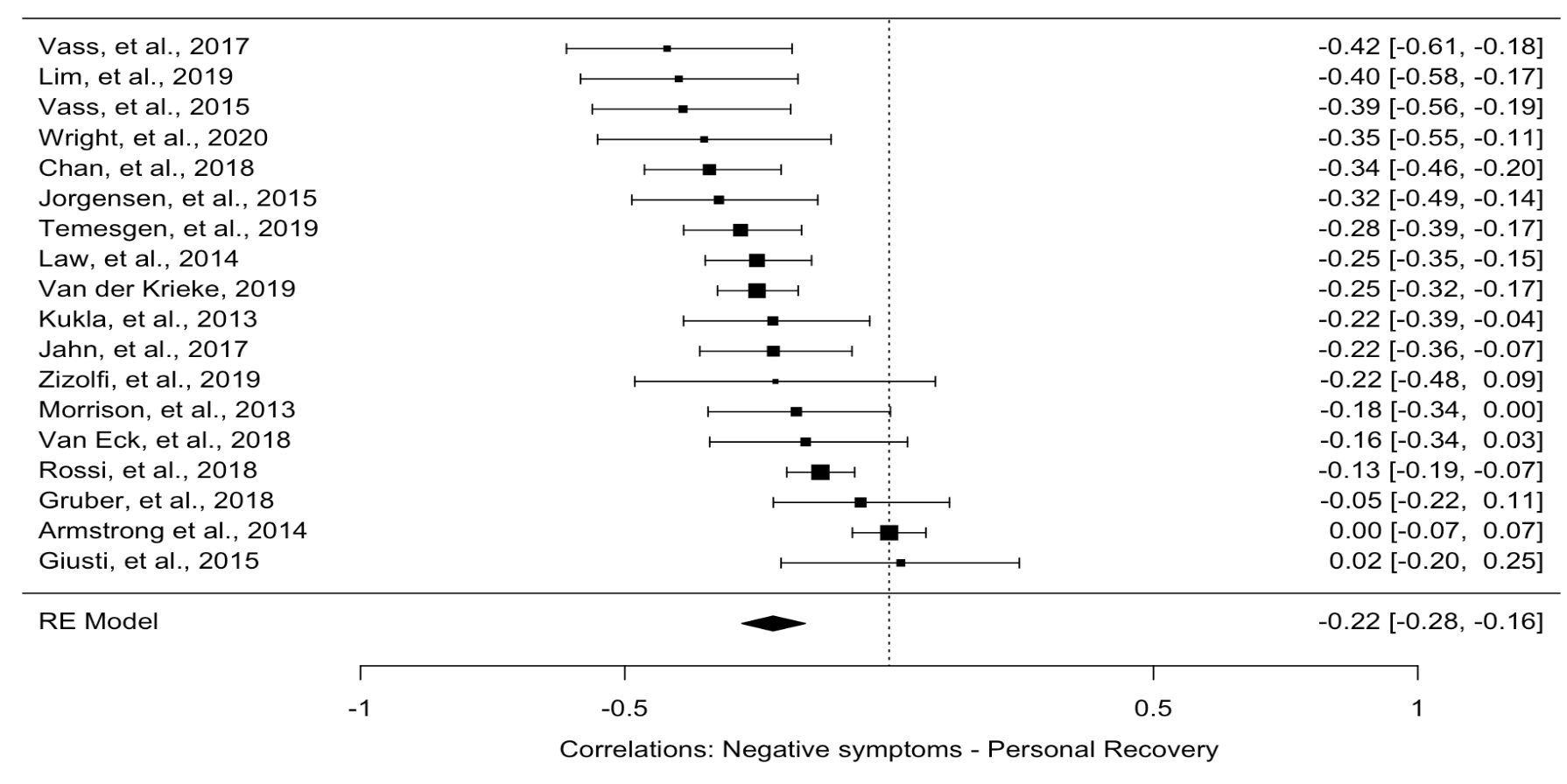


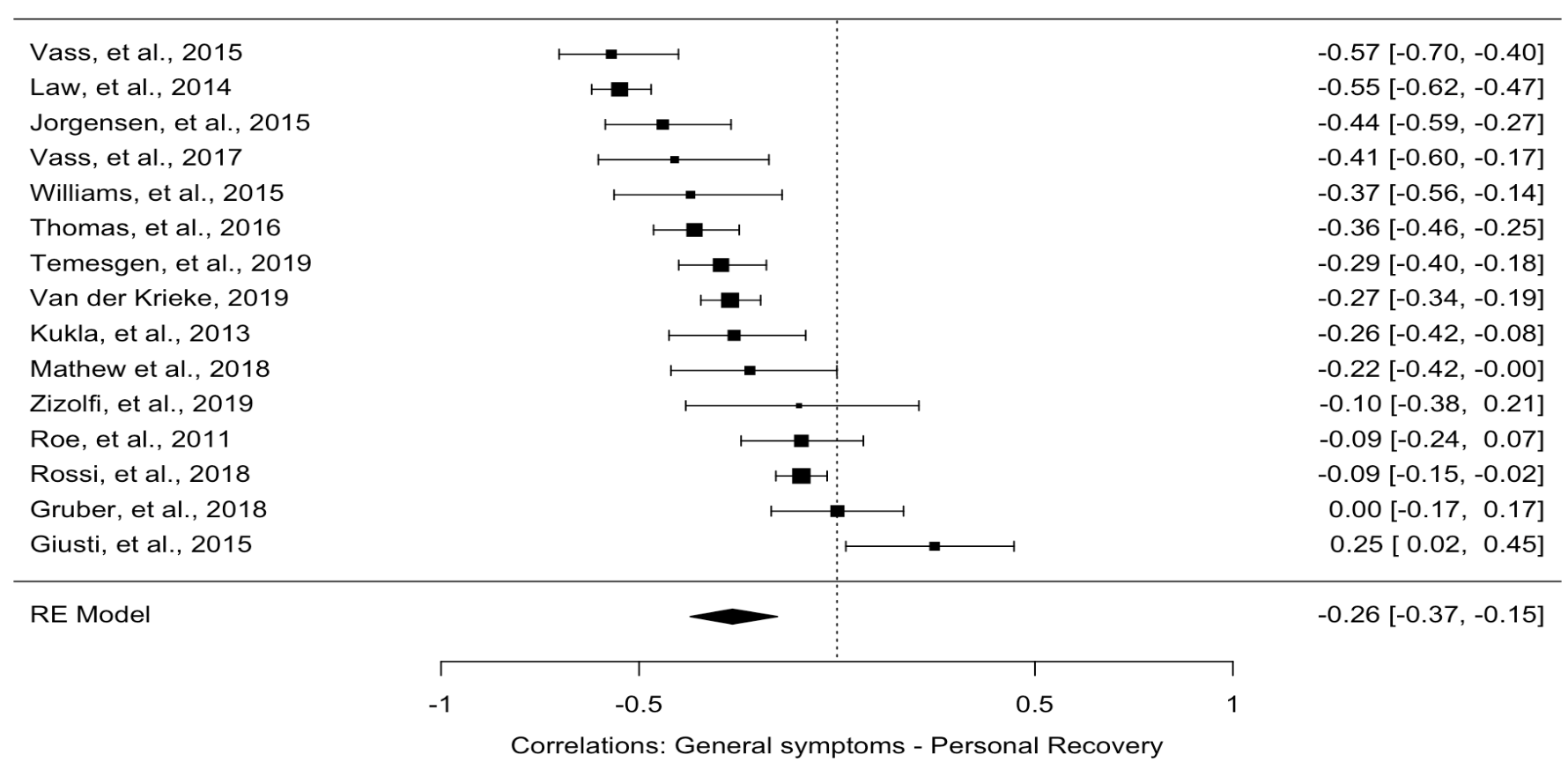


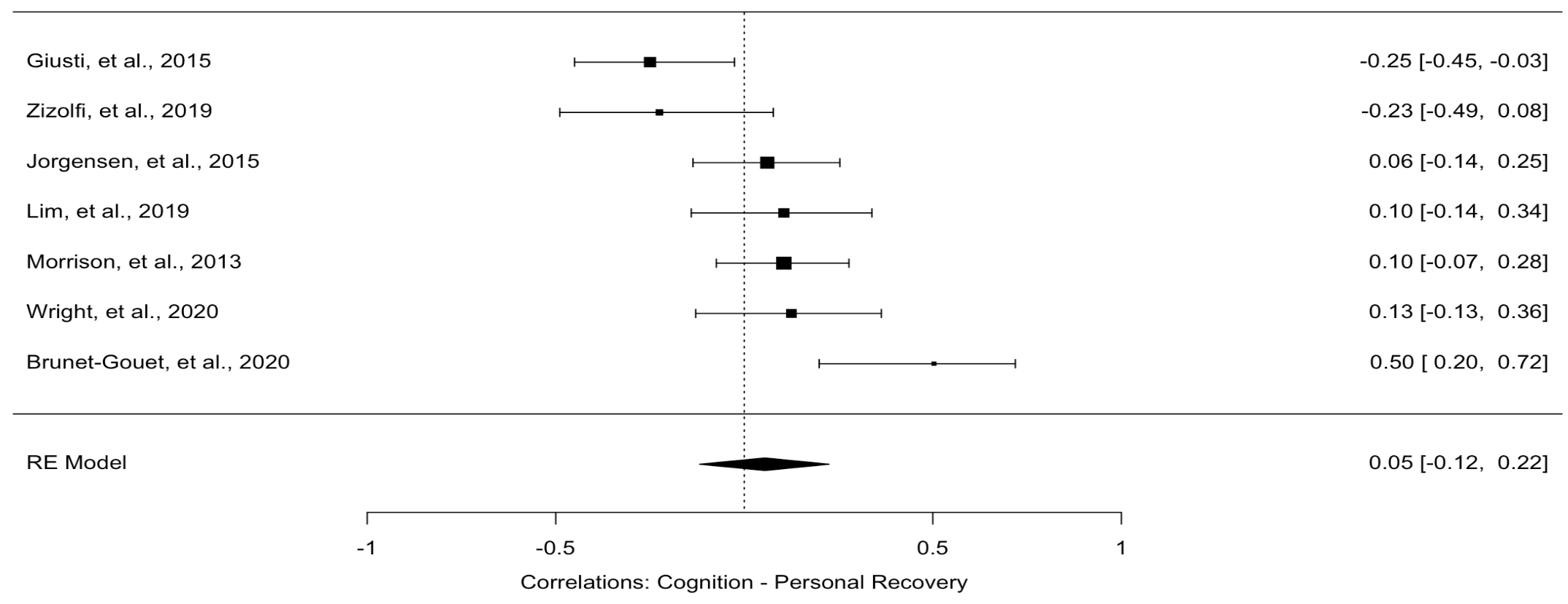


Table 2c. Forest plots of Social factors and Personal Recovery


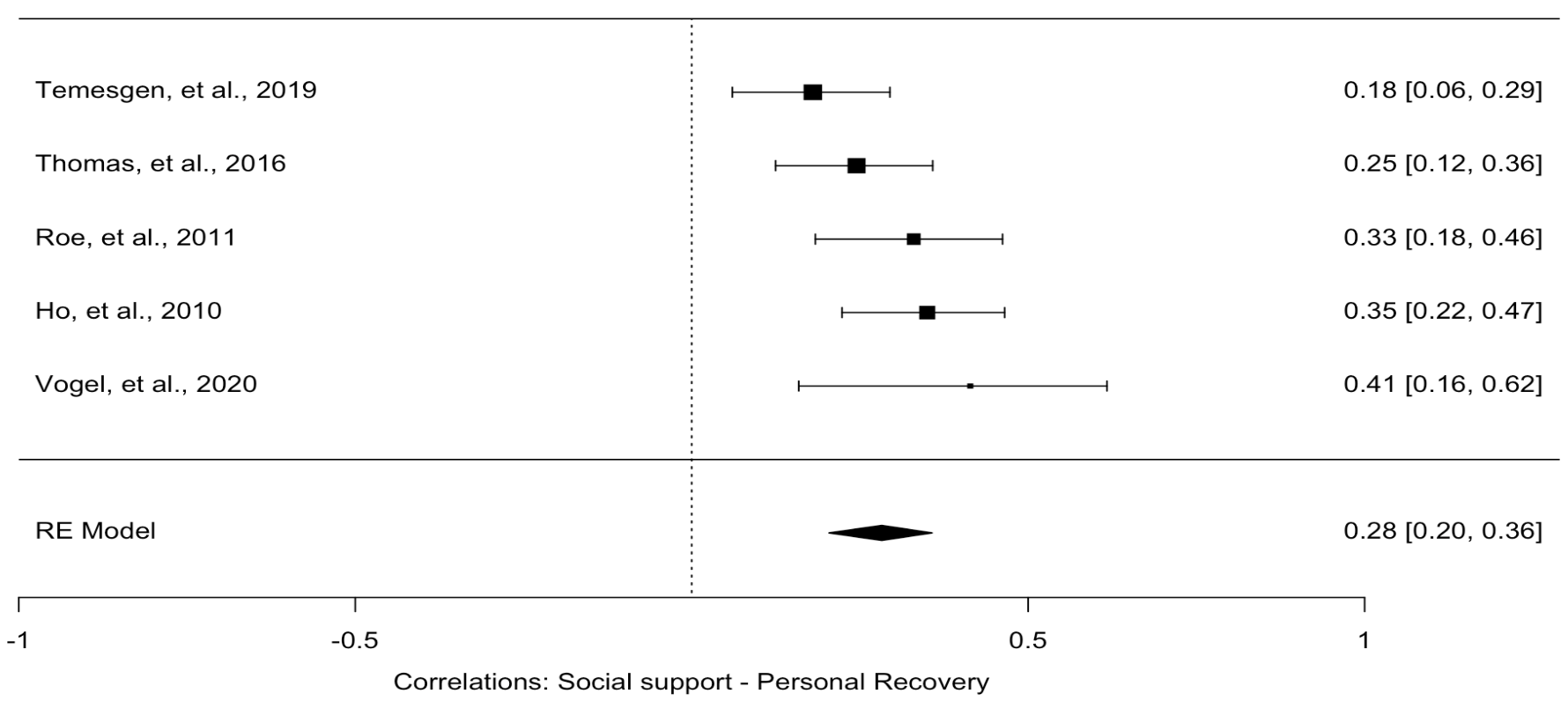


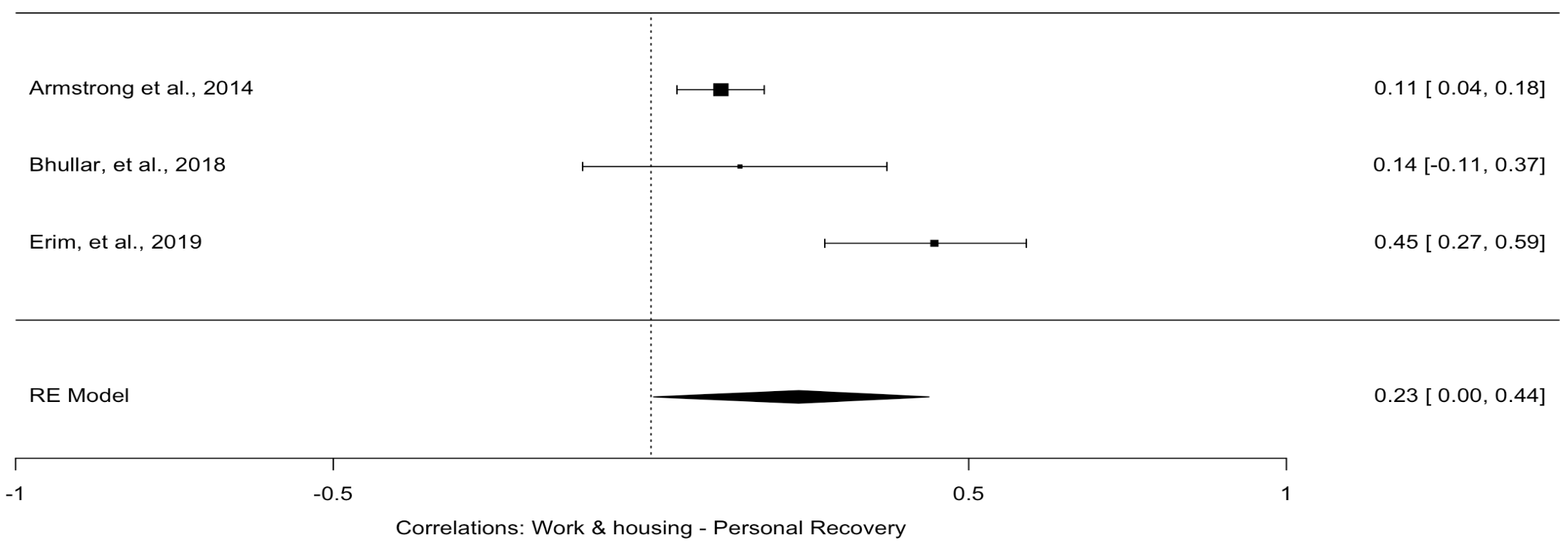


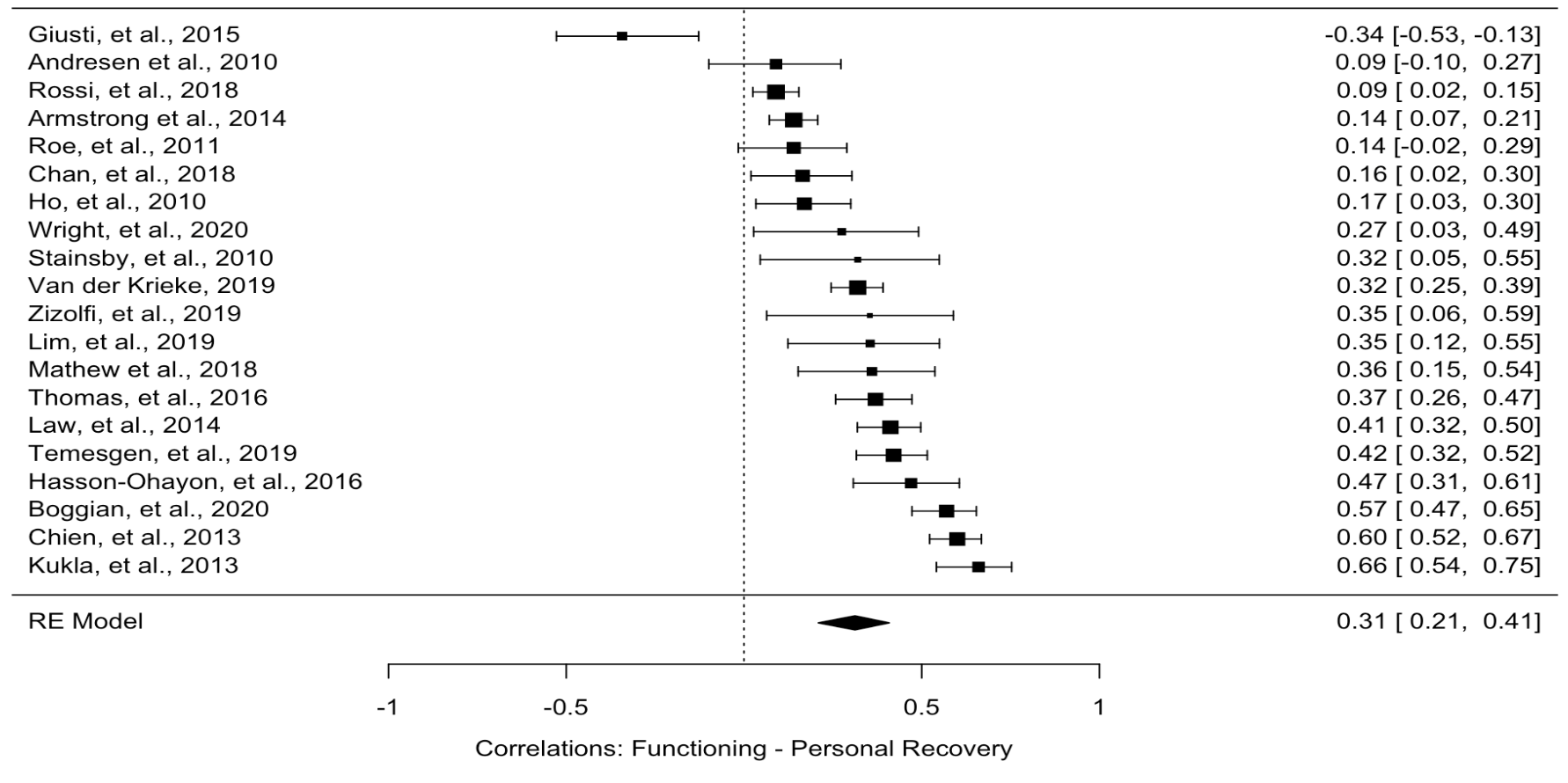

Supplement: Supplementary file 2 [file Table_2.docx]
